# Heroin Regulates the Voltage-Gated Sodium Channel Auxiliary Subunit, SCN1b, to Modulate Nucleus Accumbens Medium Spiny Neuron Intrinsic Excitability and Cue-Induced Heroin Seeking

**DOI:** 10.1523/ENEURO.0017-25.2025

**Published:** 2025-03-11

**Authors:** Ethan M. Anderson, Evgeny Tsvetkov, Daniel Wood, Rose Marie Akiki, Karim Al Hasanieh, Lauren M. McCue, Makoto Taniguchi, Antonieta Lavin, Christopher W. Cowan

**Affiliations:** ^1^Department of Neuroscience, Medical University of South Carolina, Charleston, South Carolina 29425; ^2^Department of Comparative Biomedical Sciences, Louisiana State University, Baton Rouge, Louisiana 70803

**Keywords:** intrinsic excitability, nucleus accumbens, reinstatement behavior, substance use disorder, voltage-gated sodium channel

## Abstract

Self-administration of addictive substances like heroin can couple the rewarding/euphoric effects of the drug with drug-associated cues, and opioid cue reactivity contributes to relapse vulnerability in abstinent individuals recovering from an opioid use disorder (OUD). Opioids are reported to alter the intrinsic excitability of medium spiny neurons (MSNs) in the nucleus accumbens (NAc), a key brain reward region linked to drug seeking, but how opioids alter NAc MSN neuronal excitability and the impact of altered MSN excitability on relapse-like opioid seeking remain unclear. Here, we discovered that self-administered, but not experimenter-administered, heroin reduced NAc protein levels of the voltage-gated sodium channel auxiliary subunit, SCN1b, in male and female rats. Viral-mediated reduction of NAc SCN1b increased the intrinsic excitability of MSNs, but without altering glutamatergic and GABAergic synaptic transmission. While reducing NAc SCN1b levels had no effect on acquisition of heroin self-administration or extinction learning, we observed a significant increase in cue-reinstated heroin seeking, suggesting that NAc SCN1b normally limits cue-reinstated heroin seeking. We also observed that NAc SCN1b protein levels returned to baseline following heroin self-administration, home-cage abstinence, and extinction training, suggesting that the noted reduction of NAc SCN1b during acquisition of heroin self-administration likely enhances MSN excitability and the strength of heroin–cue associations formed during active heroin use. As such, enhancing NAc SCN1b function might mitigate opioid cue reactivity and a return to active drug use in individuals suffering from OUD.

## Significance Statement

Opioid use disorder (OUD) is a chronic, relapsing disease characterized by excessive craving. Here, we found that repeated heroin self-administration reduced the expression of the sodium channel subunit, SCN1b, in the nucleus accumbens, a brain area important for reward signaling and addiction. We show here that reducing SCN1b increased the excitability of NAc neurons and increased relapse-like drug seeking in a rodent model of opioid craving. The discovery of this novel mechanism of opioid action in the brain could help lead to future treatments for patients who suffer from OUD.

## Introduction

The current opioid crisis (Volkow and Blanco, 2021) has highlighted that individuals with opioid use disorder (OUD) have difficulty remaining abstinent despite repeated attempts to discontinue their drug use. The use of addictive opioids, like heroin, often produces a strong association between the rewarding/euphoric effects and drug-associated cues in the environment. These drug use-linked associations are often enduring, and they can promote a return to active drug use in individuals recovering from an OUD and attempting to remain abstinent.

The nucleus accumbens (NAc), a region within the mesolimbic dopamine pathway, is an important area for drug reward and craving (Anderson et al., 2018; Anderson and Taniguchi, 2022). Opioids act on the NAc, both directly and indirectly, to produce adaptations that influence the development and persistence of OUD and drug–cue associations. One noted neuroadaptation is an opioid-induced change in the intrinsic excitability of NAc medium spiny neurons (MSNs), which occurs following acute (Ma et al., 2012; Trieu et al., 2022) or repeated opioid exposure (Heng et al., 2008; Wu et al., 2012; Wu et al., 2013; McDevitt et al., 2019), and changes in MSN intrinsic properties have been linked to drug-related behaviors (Dong et al., 2006), but the relationship between NAc MSN intrinsic excitability and heroin seeking remains unclear.

The sodium channel subunit SCN1b (aka, Navβ1, Navb1) is a single-pass transmembrane protein that serves as an auxiliary subunit of pore-forming voltage-gated Na^+^ channels that contribute to the rising phase of action potentials (Calhoun and Isom, 2014; Al-Ward et al., 2020). SCN1b is enriched at axon initial segments (Wimmer et al., 2015), and it modulates the intrinsic excitability of neurons (Aman et al., 2009; Patino et al., 2009; Marionneau et al., 2012; Hull et al., 2020). This hyperexcitability state likely explains why SCN1b null mice have severe seizures (Chen et al., 2004; Brackenbury et al., 2013) and why mutations in *SCN1B* are linked to epilepsy (Wallace et al., 1998; Wallace et al., 2002; Scheffer et al., 2007), cardiac arrythmia (Lopez-Santiago et al., 2007), and pain (Lopez-Santiago et al., 2011; Calhoun and Isom, 2014). In addition to diseases involving altered neuronal excitability, studies examining SCN1b in the prefrontal and ventral midbrain of mice exposed to alcohol reported that *SCN1B* was an ethanol-responsive gene (Wolen et al., 2012), and in human postmortem tissue, *SCN1B* mRNA was downregulated in the ventral midbrain of individuals that died from a cocaine overdose (Bannon et al., 2014). However, the functional link between SCN1b and substance use disorders is unknown. Moreover, while SCN1b is expressed in NAc neurons, including MSNs (Saunders et al., 2018), its role and regulation of MSN intrinsic excitability and drug-seeking behavior are unexplored. In this study, we found that heroin self-administration reduced SCN1b protein levels in the adult rat NAc. Viral-mediated reduction of SCN1b in NAc increased the intrinsic excitability of NAc MSNs, which had no effect on active heroin self-administration or extinction learning, but augmented cue-induced heroin seeking, suggesting that heroin-induced reduction of SCN1b in NAc during active self-administration serves to promote the strength of heroin–cue associations and increases future relapse-like heroin seeking.

## Materials and Methods

### Animals and animal care

Male and female adult Sprague Dawley rats were singly housed in a climate-controlled environment (21°C) on a 12 h light/dark cycle. All rats were habituated to the housing environment for at least 7 d prior to use in experiments. Rats had food and water *ad libitum* except during the acquisition of heroin self-administration as detailed below. All behavioral experiments were performed during the dark cycle and were approved by the MUSC Institutional Animal Care and Use Committee (IACUC) in facilities accredited by the American Association for the Accreditation of Laboratory Animal Care (AAALAC). All procedures were conducted in accordance with the guidelines established by the National Institutes of Health and the National Research Council.

### Heroin or saline self-administration for tissue collection

Rats underwent heroin or saline self-administration, as described previously (Anderson et al., 2023). In brief, a chronic indwelling jugular vein catheter was implanted, and ketophen (5 mg/kg) was used for postsurgical pain relief. All self-administration experiments occurred in operant chambers with two retractable levers, a house light, and a cue light and tone generator (Med Associates). During 3 h sessions on a fixed ratio 1 (FR1) schedule, rats were allowed to self-administer either saline or heroin hydrochloride (100 µg/infusion for Days 1–2, 50 µg/infusion for Days 3–4, and then 25 µg/infusion starting on Day 5). Compound tone and light cues were activated during the infusions. Patency was regularly ensured via methohexital infusions into the catheter. NAc tissues were harvested bilaterally immediately after the 12th saline or heroin self-administration session.

In a different set of rats, we repeated heroin or saline self-administration for 12 d. Following forced abstinence in their home cages for 7 d, all rats began extinction training for 6 d. During extinction training, “paired” lever presses produced no drug infusion or light/tone cues. The following day, NAc tissues were harvested bilaterally.

### Heroin or saline experimenter administration for tissue collection

Rats were injected with either saline or heroin (2 mg/kg, i.p.) for 12 consecutive days. This dose is similar to the amount of heroin self-administered by rodents during a normal 3 h session. Thirty minutes following their last dose, NAc tissues were harvested bilaterally.

### Immunoblotting

NAc tissue was isolated, lysed, and immunoblotted according to previously published methods (Anderson et al., 2021). In brief, samples were homogenized using a tissue lysis buffer [5 mM HEPES (pH 7.4), 1% (v/v) sodium dodecyl sulfate (SDS), 320 mM sucrose, 10 mM NaF, and protease inhibitors in milliQ H_2_O]. Protein concentration was estimated using the Bio-Rad DC Protein Assay. Subsequently, 20 µg/µl of protein was run on 4–20% TGX precast gradient gels (Bio-Rad). The gels were then transferred onto a Bio-Rad Trans-Blot Turbo Transfer Pack (midi format, 0.20 µm PVDF, catalog #1704157) using the Bio-Rad Trans-Blot Turbo Transfer System and placed in Odyssey blocking solution for 1 h. Primary antibody: anti-SCN1b (RRID:AB_2341071, catalog #ASC-041, Alomone Labs, rabbit, 1:1,000) and anti-tubulin beta 3 Tuj1 (RRID:AB_10063408, catalog #801202, BioLegend, mouse, 1:50,000). Secondary antibody: 800CW anti-mouse (RRID:AB_621842, catalog #926-32210, LI-COR, goat, 1:10,000) or 680CW anti-rabbit (RRID:AB_10956166, catalog 926-68071, LI-COR, goat, 1:10,000). Blots were developed on a LI-COR Odyssey CLx and analyzed with ImageStudio or ImageJ.

### Viral vectors

Neurotropic viral-mediated SCN1b reduction in the rat NAc was accomplished using a novel short-hairpin RNA interference strategy [AAV2-shSCN1b; sequence: CGACTACGAATGTCACGTCTA; University of South Carolina (USC) Viral Vector Core], similar to published studies (Anderson et al., 2023). The control virus was a short hairpin to luciferase with no known homology in the rat (AAV2-shLuc; sequence: CTTACGCTGAGTACTTCGA, USC Viral Vector Core). Both shRNA viruses coexpress EGFP under control of a CMV promoter.

### Stereotaxic surgery

Rats underwent isoflurane-anesthetized survival surgery to microinject 1.0 µl of viral vector bilaterally into the NAc (AP, +1.7; DV, −7.7; ML, ±1.0; no angle). These coordinates target both the core and shell regions. All rats in the study were allowed at least 5 d of recovery before any additional experimentation. Ketophen (5 mg/kg) was used for postsurgical pain relief.

### Quantitative RT-PCR

mRNA was extracted using QIAzol Lysis Reagent (Qiagen, catalog #56008534) and the RNeasy Micro Kit (Qiagen, catalog #74004). cDNA libraries were made with a SuperScript 3 kit (Invitrogen). qRT-PCR was performed using a Bio-Rad CFX96 using the following primer sets: Scn1b (forward: ACGTGCTCATTGTGGTGTTAACC; reverse: CCGTGGCAGCAGCAATC) and normalized to Gapdh (forward: AGGTCGGTGTGAACGGATTTG; reverse: TGTAGACCATGTAGTTGAGGTCA).

### Immunohistochemistry (IHC)

Following an anesthetized transcardial perfusion with 4% (w/v) paraformaldehyde in 1× PBS and a 24 h postfix at 4°C, brains were cryoprotected with 30% (w/v) sucrose before generation of 60 µm frozen slices. Coronal slices were then blocked [3% (w/v) bovine serum albumen, 1.5% (v/v) normal donkey serum, 0.2% (v/v) Triton X-100, 0.2% (v/v) Tween 20 in PBS] for at least 1 h and then transferred to new blocking buffer containing anti-GFP (RRID: AB_10000240, catalog #GFP-1020, Aves Labs, chicken, 1:4,000) and incubated at 4°C overnight with slow agitation. The next day, tissue was washed 3 × 5 min in PBS before 1 h incubation with anti-chicken secondary antibody (RRID: AB_2340375, catalog #703-545-155, Alexa Fluor 488 donkey anti-chicken, The Jackson Laboratory, 1:500). Slices were then washed in bisbenzimide (1:5,000, Hoechst 33342, Invitrogen) for 2 min, followed by 2 × 5 min PBS washes and then mounted on slides. Images were acquired with a Leica Thunder 3D Tissue Imager and processed with ImageJ (RRID: SCR_002285, Fiji, NIH).

### Electrophysiology

All acute-slice electrophysiological experiments were performed in Sprague Dawley rats at least 3 weeks after an AAV surgery. Acute coronal slices (300 µm thickness) containing the NAc were prepared in a semifrozen artificial cerebrospinal fluid (ACSF) containing the following (in mM): 127 NaCl, 2.5 KCl, 1.2 NaH_2_PO_4_, 24 NaHCO_3_, 11 D-glucose, 1.2 MgCl_2_, 2.40 CaCl_2_, and 0.4 Na-ascorbate (pH 7.4, 315–320 mOsm). Kynurenic acid (5 mM) was added to ACSF to avoid overactivity of glutamatergic receptors during slicing. Slices were transferred to ACSF without kynurenic acid to recover at 37°C for 30 mins and then transferred to room temperature ACSF for an additional 30 min prior to recording. All solutions were continually equilibrated with 95% O_2_ and 5% CO_2_ prior to and during the slicing procedure. Experiments were performed in whole-cell clamp mode using electrodes with a resistance of 4–6 MΩ pulled using a Narishige puller (Narishige, PG10) from borosilicate tubing (Sutter Instrument) and filled by an internal solution containing the following (in mM): 140 CsMetSO_4_, 5 KCl, 1 MgCl_2_, 0.2 EGTA, 11 HEPES, 2 NaATP, 0.2 Na_2_GTP, and 0.1 CaCl_2_ (pH 7.2, 290–295 mOsm) for voltage-clamp experiments or 120 K-gluconate,10 NaCl, 10 KCl, 0.5 EGTA, 2 Na_2_ATP, 0.3 Na-GTP, 10 HEPES, and 10 phosphocreatine (pH 7.2–7.35 and 270–280 mOsm) for current-clamp experiments. Intrinsic excitability was recorded via whole-cell current-clamp mode, and the transmembrane current was clamped, at −70 mv. The current was injected at 20 pA steps starting at 0 pA. Each step was held for 1,000 ms, and current/frequency curves were generated. Any neuron that didn't show spikes or only spiked once across the 0–500 pA range of currents was removed from the analysis. Amplitude accommodation was analyzed using MiniAnalysis. The window for decimation was first set to the peak of the second spike, and if the subsequent spikes were smaller (in descending order) than the second spike, the cell was considered to exhibit spike amplitude accommodation. Action potential properties including amplitude, rise time, rise time of 10–90, decay, and half-width were analyzed using MiniAnalysis. AMPA and NMDA receptor-mediated excitatory postsynaptic currents (AMPA and NMDA responses) were recorded respectively at −70 and +50 mV in voltage-clamp mode. Recordings were made in the presence of picrotoxin (50 µM) to block inhibitory postsynaptic currents mediated by GABA_A_ receptors. The amplitude of AMPA responses was calculated at the maximum current value, and the amplitude of the NMDA response was calculated at 50 ms poststimulation. These values were used to calculate the AMPA/NMDA ratio. We also measured the peak amplitude and half-width of both AMPA and NMDA responses. For paired-pulse ratio (PPR) measurements, two EPSCs were evoked at −70 mV with an interstimulus interval of 50 ms. The peak amplitude of the second EPSC (P2) was divided by the peak of the first amplitude (P1) to generate the PPR (P2/P1). All recording data were acquired and analyzed by amplifier Axopatch 200B (Axon Instruments), digitizer BNC2090 (National Instruments), and software AxoGraph v1.7.0, Clampfit v8.0 (pClamp, Molecular Devices), and MiniAnalysis Program v6.0.9 (Synaptosoft). Data were filtered at 2 kHz by Axopatch 200B amplifier (Axon Instruments) and digitized at 20 kHz via AxoGraph v1.7.0.

### Heroin self-administration, extinction training, and cue-induced reinstatement

Following a viral infusion, rats underwent a 2-week incubation period before catheterization and acquisition of heroin self-administration as described above. Rats were allowed to self-administer heroin until they reached a criterion of at least 10 infusions of heroin for at least 12 d. After a 7 d home-cage abstinence period, rats began extinction training for at least 6 d and until a criterion of fewer than 25 presses per session for 2 consecutive days was met. During extinction training, “paired” lever presses produced no drug infusion or light/tone cues. Next, cue-induced reinstatement behavior was tested on the following day by allowing the rats to respond to light and tone cues without receiving heroin injections for a single 3 h session (Anderson et al., 2023). Rats were perfused under anesthesia following the behavioral experiments, and accuracy of viral targeting to the NAc was assessed by the presence of GFP following slicing as described above. Only rats with bilateral NAc-targeted viral GFP expression were included in the final analysis.

### Statistics

*t* tests and repeated-measures two-way ANOVAs were used to analyze data where appropriate. Fisher’s LSD post hoc tests were used following significant interactions using ANOVAs. Grubbs' test was used to identify single statistical outliers. A chi-square analysis was used to analyze differences in amplitude accommodation. All statistics were performed with GraphPad Prism 9, and *p* < 0.05 was considered significant. All statistics are reported in Table 1, including when a Grubbs' test found a significant outlier.

**Table 1.** Statistics for Anderson et al.

		Test	Groups	*N* (rats)	*p*-value		Grubbs’ test
Figure 1	B	RM two-way ANOVA (interaction)	Saline versus heroin (infusions)	(7,7)	<0.0001	*F*_(11,132)_ *=* 7.969	
	B	RM two-way ANOVA (time)	Saline versus heroin (infusions)	(7,7)	0.1029	*F*_(4.358,52.30)_ *=* 1.998	
	B	RM two-way ANOVA (drug)	Saline versus heroin (infusions)	(7,7)	0.6352	*F*_(1,12)_ *=* 0.2370	
	C	RM two-way ANOVA (interaction)	Saline versus heroin (paired lever presses)	(7,7)	<0.0001	*F*_(11,132)_ *=* 5.900	
	C	RM two-way ANOVA (time)	Saline versus heroin (paired lever presses)	(7,7)	0.1536	*F*_(2.948,35.38)_ *=* 1.8688	
	C	RM two-way ANOVA (drug)	Saline versus heroin (paired lever presses)	(7,7)	0.0146	*F*_(1,12) _*=* 8.128	
	D, left	*t* test	Self-administered saline versus heroin (SCN1b/beta-tubulin protein)	(7,7)	0.0113	*t* *=* 2.987, df *=* 12	
	D, right	*t* test	Experimenter-administered saline versus heroin (SCN1b/beta-tubulin)	(8,8)	0.9861	*t* *=* 0.01771, df *=* 14	
	G	*t* test	AAV-shLUC versus AAV-shSCN1b	(3,3)	0.0096	*t* *=* 4.659, df *=* 4	
						
Figure 2	C	*t* test	AAV-shLUC versus AAV-shSCN1b—rheobase	(19/8, 16/6)	0.0002	*t* *=* 4.153, df *=* 33	One outlier removed
	D	RM two-way ANOVA (interaction)	AAV-shLUC versus AAV-shSCN1b—spiking	(19/8, 17/6)	0.1783	*F*_(14,476)_ *=* 1.342	
	D	RM two-ay ANOVA (time)	AAV-shLUC versus AAV-shSCN1b—spiking	(19/8, 17/6)	<0.0001	*F*_(2.653,90.22)_ *=* 23.20	
	D	RM two-way ANOVA (virus)	AAV-shLUC versus AAV-shSCN1b—spiking	(19/8, 17/6)	0.4492	*F*_(1,34)_ *=* 0.5862	
	F	*t* test	AAV-shLUC versus AAV-shSCN1b—amplitude	(19/8, 17/6)	0.0149	*t* *=* 2.565, df *=* 34	
	G	*t* test	AAV-shLUC versus AAV-shSCN1b—rise time	(18/8, 17/6)	0.0059	*t* *=* 2.944, df *=* 33	One outlier removed
	H	*t* test	AAV-shLUC versus AAV-shSCN1b—decay	(18/8, 17/6)	0.0950	*t* *=* 1.719, df *=* 33	One outlier removed
	J	*t* test	AAV-shLUC versus AAV-shSCN1b—AMPA/NMDA ratio	(15/5,18/5)	0.9820	*t* *=* 0.02268, df=31	One outlier removed
	L	*t* test	AAV-shLUC versus AAV-shSCN1b—paired-pulse ratio	(7/3,12/3)	0.3048	*t* *=* 1.058, df *=* 17	One outlier removed
						
Figure 3	B	RM two-way ANOVA (interaction)	AAV-shLUC versus AAV-shSCN1b—infusions	(8,9)	0.8629	*F*_(11,165)_ *=* 0.5548	
	B	RM two-way ANOVA (time)	AAV-shLUC versus AAV-shSCN1b—infusions	(8,9)	0.0001	*F*_(11,165)_ *=* 21.78	
	B	RM two-way ANOVA (virus)	AAV-shLUC versus AAV-shSCN1b—infusions	(8,9)	0.8987	*F*_(1,15)_ *=* 0.01676	
	C	RM two-way ANOVA (interaction)	AAV-shLUC versus AAV-shSCN1b—paired lever presses	(8,9)	0.8693	*F*_(11,165)_ *=* 0.5462	
	C	RM two-way ANOVA (time)	AAV-shLUC versus AAV-shSCN1b—paired lever presses	(8,9)	0.0001	*F*_(11,165) _*=* 7.469	
	C	RM two-way ANOVA (virus)	AAV-shLUC versus AAV-shSCN1b—paired lever presses	(8,9)	0.6782	*F*_(1,15)_ *=* 0.1790	
	C	RM two-way ANOVA (interaction)	AAV-shLUC versus AAV-shSCN1b—unpaired lever presses	(8,9)	0.6192	*F*_(11,165)_ *=* 0.8210	
	C	RM two-way ANOVA (time)	AAV-shLUC versus AAV-shSCN1b—unpaired lever presses	(8,9)	0.7658	*F*_(11,165)_ *=* 0.6696	
	C	RM two-way ANOVA (virus)	AAV-shLUC versus AAV-shSCN1b—unpaired lever presses	(8,9)	0.9014	*F*_(1,15) _*=* 0.01587	
	D	RM two-way ANOVA (interaction)	AAV-shLUC versus AAV-shSCN1b—discrimination ratio	(8,9)	0.7076	*F*_(11,165) _*=* 0.7310	
	D	RM two-way ANOVA (time)	AAV-shLUC versus AAV-shSCN1b—discrimination ratio	(8,9)	<0.0001	*F*_(11,165) _*=* 5.414	
	D	RM two-way ANOVA (virus)	AAV-shLUC versus AAV-shSCN1b—discrimination ratio	(8,9)	0.6216	*F*_(1,15)_ *=* 0.2539	
	E	RM two-way ANOVA (interaction)	AAV-shLUC versus AAV-shSCN1b—time-out responding	(8,9)	0.8836	*F*_(11,165) _*=* 0.5262	
	E	RM two-way ANOVA (time)	AAV-shLUC versus AAV-shSCN1b—time-out responding	(8,9)	0.0001	*F*_(11,165)_ *=* 5.920	
	E	RM two-way ANOVA (virus)	AAV-shLUC versus AAV-shSCN1b—time-out responding	(8,9)	0.6500	*F*_(1,15)_ *=* 0.2144	
	F	RM two-way ANOVA (interaction)	AAV-shLUC versus AAV-shSCN1b—extinction paired lever presses	(8,9)	0.9450	*F*_(5,75)_ *=* 0.2370	
	F	RM two-way ANOVA (time)	AAV-shLUC versus AAV-shSCN1b—extinction paired lever presses	(8,9)	0.0001	*F*_(5,75)_ *=* 21.05	
	F	RM two-way ANOVA (virus)	AAV-shLUC versus AAV-shSCN1b—extinction paired lever presses	(8,9)	0.5487	*F*_(1,15)_ *=* 0.3765	
	F	RM two-way ANOVA (interaction)	AAV-shLUC versus AAV-shSCN1b—extinction unpaired lever presses	(8,9)	0.9883	*F*_(5,75)_ *=* 0.1170	
	F	RM two-way ANOVA (time)	AAV-shLUC versus AAV-shSCN1b—extinction unpaired lever presses	(8,9)	0.0001	*F*_(5,75) _*=* 12.19	
	F	RM two-way ANOVA (virus)	AAV-shLUC versus AAV-shSCN1b—extinction unpaired lever presses	(8,9)	0.5620	*F*_(1,15)_ *=* 0.3517	
	G	RM two-way ANOVA (interaction)	AAV-shLUC versus AAV-shSCN1b—cue-induced reinstatement	(8,9)	0.0471	*F*_(1,15)_ *=* 4.680	
	G	RM two-way ANOVA (time)	AAV-shLUC versus AAV-shSCN1b—cue-induced reinstatement	(8,9)	0.0002	*F*_(1,15)_ *=* 23.85	
	G	RM two-way ANOVA (virus)	AAV-shLUC versus AAV-shSCN1b—cue-induced reinstatement	(8,9)	0.0957	*F*_(1,15)_ *=* 3.160	
	G	RM two-way ANOVA (virus)	Post hoc test following a significant interaction	(8,9)	0.0096		
	H	RM two-way ANOVA (interaction)	Saline versus heroin (infusions)	(6,10)	<0.0001	*F*_(11,154)_ *=* 10.25	
	H	RM two-way ANOVA (time)	Saline versus heroin (infusions)	(6,10)	0.3044	*F*_(2.653,37.15)_ *=* 1.249	
	H	RM two-way ANOVA (drug)	Saline versus heroin (infusions)	(6,10)	0.2161	*F*_(1,14)_ *=* 1.678	
	I	RM two-way ANOVA (interaction)	Saline versus heroin (paired lever presses)	(6,10)	0.0001	*F*_(11,154)_ *=* 3.646	
	I	RM two-way ANOVA (time)	Saline versus heroin (paired lever presses)	(6,10)	0.5600	*F*_(2.326,32.56)_ *=* 0.6337	
	I	RM two-way ANOVA (drug)	Saline versus heroin (paired lever presses)	(6,10)	0.1985	*F*_(1,14)_ *=* 1.822	
	J	RM two-way ANOVA (interaction)	Saline versus heroin—extinction paired lever presses	(6,10)	<0.0001	*F*_(5,70)_ *=* 7.239	
	J	RM two-way ANOVA (time)	Saline versus heroin—extinction paired lever presses	(6,10)	<0.0001	*F*_(2.047,28.66)_ *=* 12.77	
	J	RM two-way ANOVA (drug)	Saline versus heroin—extinction paired lever presses	(6,10)	0.0019	*F*_(1,14)_ *=* 14.45	
	K	*t* test	Saline versus heroin plus extinction (SCN1b/beta-tubulin protein)	(6,10)	0.2763	*t* *=* 1.148, df *=* 14	

## Results

### NAc SCN1b protein levels are reduced by heroin self-administration, but not by experimenter-administered heroin

**Figure 1.**
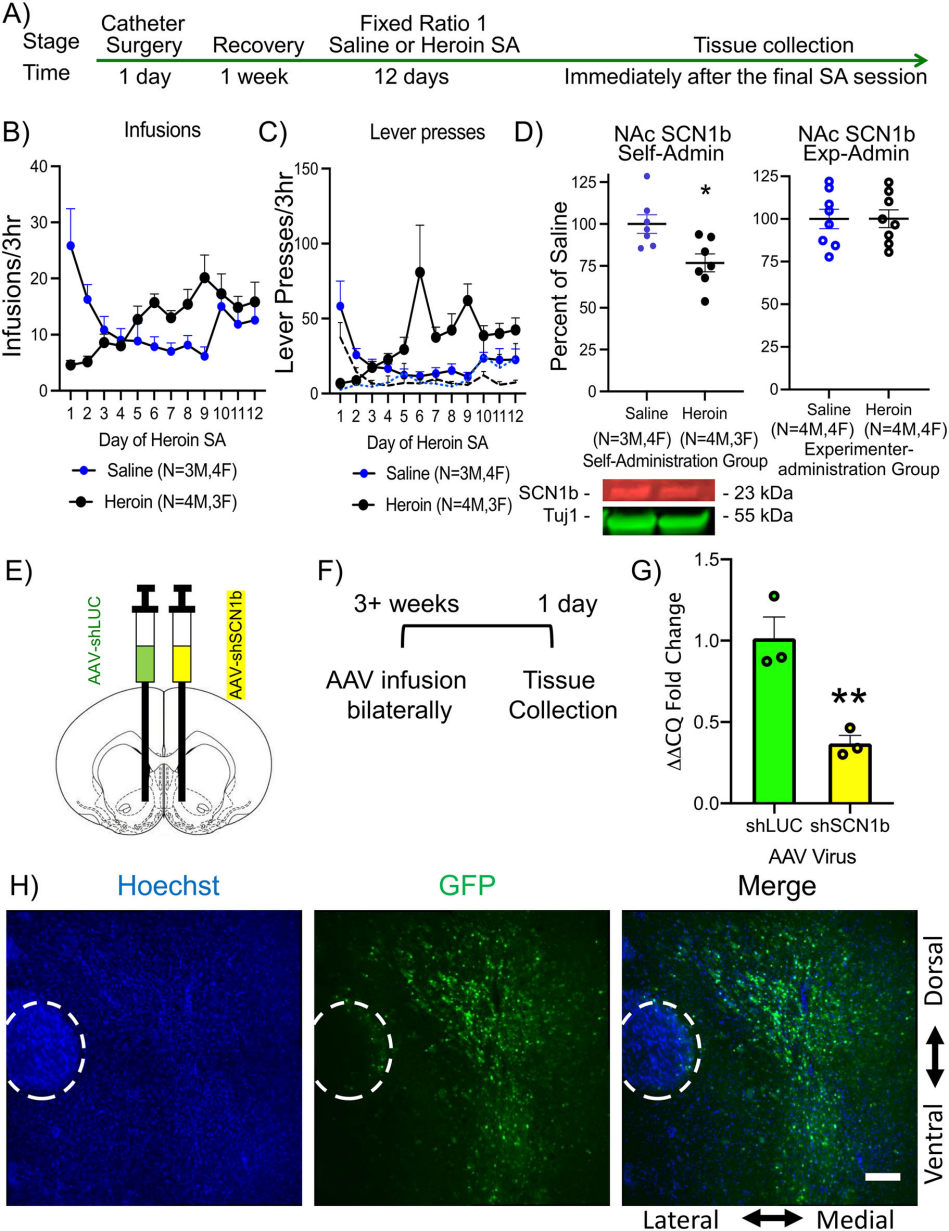
Heroin self-administration reduced NAc SCN1b, and AAV-shSCN1b mimicked this effect. ***A***, Experimental timeline for tissue collection. ***B***, Heroin versus saline infusions and (***C***) paired (solid lines) versus unpaired lever presses (dotted lines) from self-administering rats. ***D***, Left, Immunoblotting showed that heroin self-administration reduced NAc SCN1b protein. ***D***, Right, In contrast, immunoblotting showed that experimenter-administered heroin had no effect on NAc SCN1b protein. ***E***, A diagram representation of the NAc stereotaxic injections and an (***F***) experimental timeline for AAV characterization. ***G***, Quantitative RT-PCR results demonstrated that AAV-shSCN1b reduced NAc SCN1b mRNA. ***H***, Representative images of AAV-shSCN1b expression in the NAc following IHC for coexpressed GFP. Scale bar, 150 um. The circled region is the anterior commissure. Data are expressed as mean ± SEM. *p < 0.05, **p < 0.01.

Rats were allowed to self-administer either heroin or saline for 12 d (Fig. 1*A*). Heroin self-administering rats increased their drug-paired lever presses and heroin infusions over subsequent days, whereas the saline self-administering rats reduced their paired lever presses and saline infusions over the same timeframe, as expected (Fig. 1*B*,*C*). At the end of the final 3 h self-administration session, bilateral NAc tissues were isolated rapidly and processed for Western blotting. Compared to rats that self-administered saline-only, we observed that NAc SCN1b levels were significantly reduced in the rats with a history of heroin self-administration (Fig. 1*D*, left). Since these effects could be due to the primary pharmacological effects of heroin, we next tested the effects of experimenter-administered saline versus heroin injections on NAc SCN1b protein levels. Following 12 d of 2 mg/kg (i.p.) heroin injections, which is similar to average doses received during a typical heroin self-administration session, we detected no significant change in NAc SCN1b protein levels (Fig. 1*D*, right). Thus, active self-administration appears to be required for a reduction of NAc SCN1b levels. To mimic the effects of heroin self-administration on NAc SCN1b, we next created a neurotropic adeno-associated virus (AAV2) expressing EGFP and a short-hairpin RNA (shRNA) targeting SCN1b mRNA for RNAi-mediated degradation (AAV2-shSCN1b). Compared to a negative control shRNA (AAV2-shLUC) virus, we observed that the AAV2-shSCN1b infused into the male rat NAc (Fig. 1*H*) significantly reduced endogenous SCN1b levels (Fig. 1*F*,*G*).

### SCN1b limits NAc MSN intrinsic excitability

**Figure 2.**
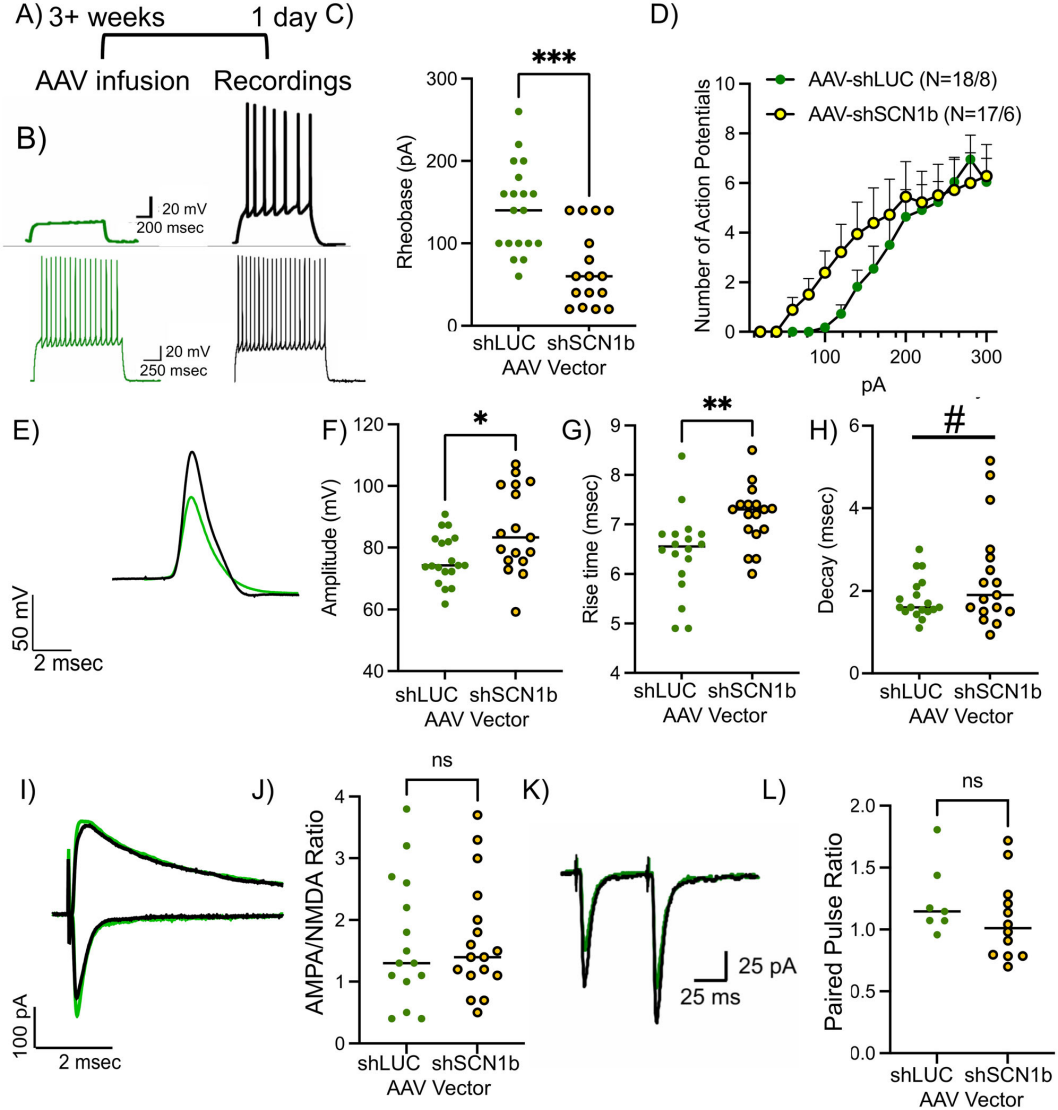
SCN1b knockdown increased intrinsic excitability in NAc MSNs. ***A***, Experimental timeline. ***B***, Top, Representative traces of control (green) versus AAV-shSCN1b NAc neurons (black) at 100 pA and ***B***, bottom, at 300 pA. ***C***, AAV-shSCN1b reduced the rheobase of NAc MSNs, but (***D***) no overall effect on excitability was observed when currents were increased. ***E***, Representative traces of an action potential from control versus AAV-shSCN1b infected neurons. ***F***, AAV-shSCN1b increased the amplitude, (***G***) rise time, and (***H***) decay of action potentials. ***I***, As shown by a representative image, (***J***) AAV-shSCN1b did not alter the AMPA/NMDA ratio. ***K***, As shown by a representative image, (***L***) AAV-shSCN1b did not alter paired-pulse ratio (50 ms interstimulus interval). Data are expressed as mean ± SEM. ^#^*p* < 0.10, *p < 0.05, **p < 0.001, and ***p < 0.0001.

Since SCN1b is reported to modulate neuronal excitability (Marionneau et al., 2012; Nguyen et al., 2012; Kubota et al., 2017), we tested the influence of SCN1b on NAc MSN intrinsic excitability. Using ex vivo acute-slice preparations in current-clamp configuration, we analyzed the number of action potentials elicited with increasing levels of current injection (Fig. 2*A*,*B*). NAc MSNs expressing AAV2-shSCN1b showed a significant decrease in rheobase (Fig. 2*B*,*C*) and a leftward shift in the current–action potential relationship at lower currents (Fig. 2*D*), indicating that endogenous SCN1b limits intrinsic excitability of NAc MSNs. The reduction of SCN1b did not alter the maximum number of action potentials generated at peak currents (Fig. 2*B*,*D*). Of note, amplitude accommodation was observed in a subset of both control (4 of 22) and shSCN1b neurons (5 of 22), but there was no difference in their occurrence between these groups [*χ*^2^ (1, *N* = 44) = 0.140, *p* = 0.7086]. These findings suggest that SCN1b does not alter amplitude accommodation. In contrast, we found that SCN1b did alter action potential kinetics (Fig. 2*E*). We observed a significant increase in the amplitude of voltage (Fig. 2*F*), rise time (Fig. 2*G*), and a trend for an increase in the decay time (Fig. 2*H*), suggesting that reducing SCN1b dysregulates sodium current kinetics and/or delays activation of voltage-gated potassium channels. Of note, AAV2-shSCN1b had no significant effects on evoked AMPA- or NMDA-mediated excitatory synaptic transmission [AMPA: AAV2-shLUC (mean = −50.13, SEM = 8.134) vs AAV2-shSCN1b (mean = −74.50, SEM = 13.39), *p* = 0.1491, *t*_(31)_ = 1.480, one outlier removed; NMDA: AAV2-shLUC (mean = 45.50, SEM = 8.603) vs AAV2-shSCN1b (mean = 55.44, SEM = 13.61), *p* = 0.5526, *t*_(32)_ = 0.6002] or the AMPA/NMDA ratio (Fig. 2*I*,*J*). In addition, no effects on activation kinetics of AMPA nor NMDA EPSCs were observed as we saw no differences in the peak amplitude of AMPA [AAV2-shLUC (mean = −0.1179, SEM = 0.02182, one outlier removed) vs AAV2-shSCN1b (mean = −0.1663, SEM = 0.04157, one outlier removed), *p* = 0.3278, *t*_(32)_ = 0.9938], the peak amplitude of NMDA [AAV2-shLUC (mean = 0.1951, SEM = 0.04131, one outlier removed) vs AAV2-shSCN1b (mean = 0.1172, SEM = 0.01243, one outlier removed), *p* = 0.0965, *t*_(32)_ = 1.7122], the half-width of AMPA [AAV2-shLUC (mean = 7.077, SEM = 0.5755) vs AAV2-shSCN1b (mean = 8.029, SEM = 0.4893, *p* = 0.2136, *t*_(34)_ = 1.268)], or the half-width of NMDA [AAV2-shLUC (mean = 47.50, SEM = 7.297) vs AAV2-shSCN1b (mean = 48.95, SEM = 5.573), *p* = 0.8775, *t*_(34)_ = 0.1553]. Finally, we observed no differences in the paired-pulse ratio of the evoked excitatory postsynaptic currents (EPSC; Fig. 2*K*,*L*; 50 ms interstimulus interval). Combined, these results suggest that SCN1b in NAc MSNs does not alter basal glutamatergic synaptic transmission or presynaptic function, but it does reduce the threshold for an NAc MSN to fire an action potential.

### NAc SCN1b limits cue-reinstated heroin seeking without altering acquisition of heroin self-administration or extinction learning

**Figure 3.**
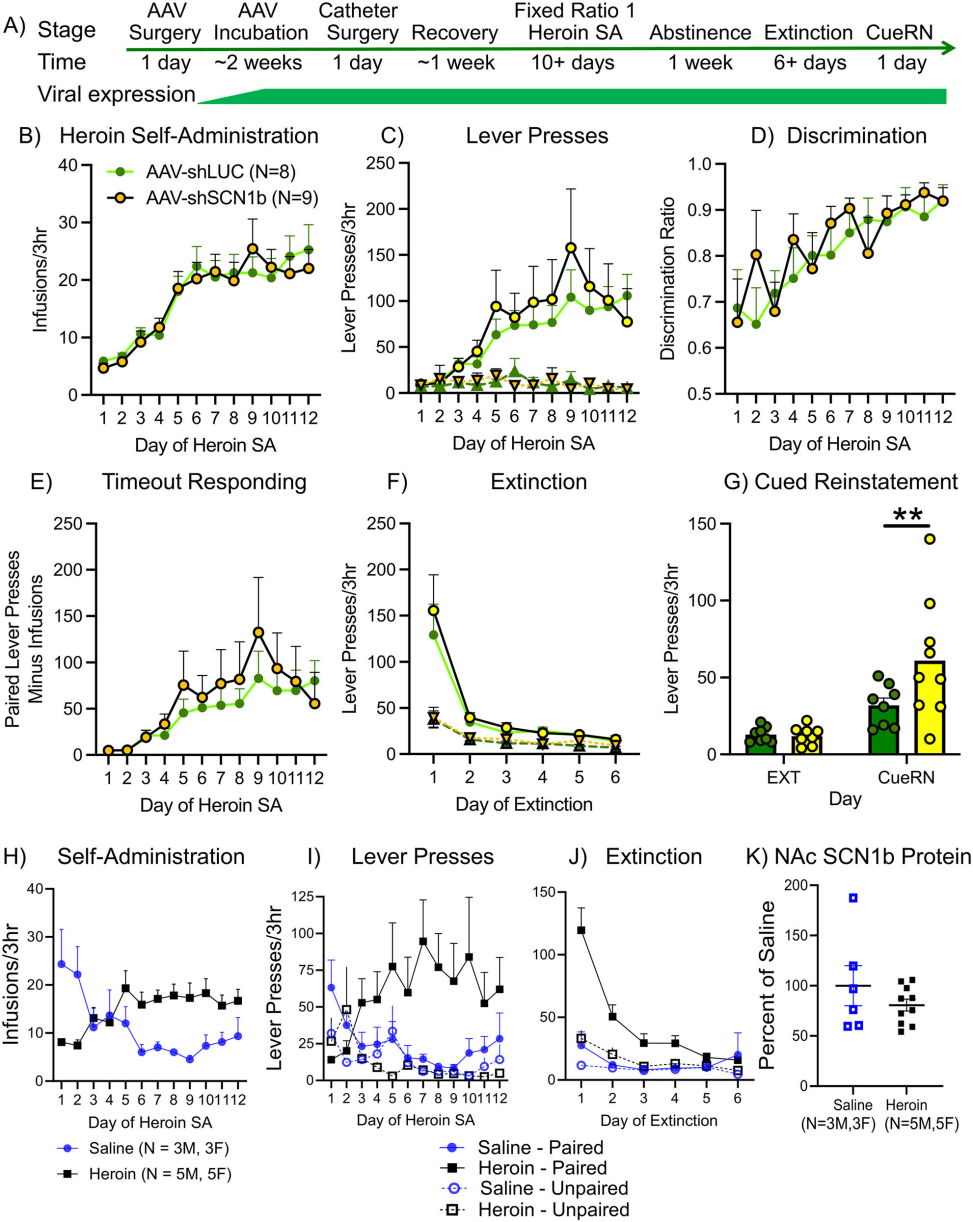
NAc SCN1b regulates cue-induced heroin-seeking behavior. ***A***, Experimental timeline. ***B***, AAV-shSCN1b had no effect on the number of infusions, (***C***) paired (solid lines) or unpaired lever presses (dotted lines), (***D***) the discrimination ratio between paired and unpaired lever presses, or (***E***) time-out responding on the paired lever during heroin self-administration. ***F***, During extinction training, AAV-shSCN1b had no effect on the number of paired (solid lines) or unpaired lever presses (dotted lines). ***G***, AAV-shSCN1b in the NAc led to increased cued-reinstatement of heroin seeking. ***H***, Heroin versus saline infusions and (***I***) paired versus unpaired lever presses from self-administering rats. ***J***, Paired versus unpaired lever presses during extinction from the same self-administering rats. ***K***, Immunoblotting showed that abstinence and extinction following heroin self-administration returned NAc SCN1b protein to baseline levels. Data are expressed as mean ± SEM. **p < 0.01.

To examine the role of NAc SCN1b on heroin self-administration behavior, we infused AAV2-shSCN1b or the shLUC control bilaterally in the adult male rat NAc and tested acquisition of heroin self-administration, extinction learning, and cue-reinstated heroin seeking (Fig. 3*A*). Viral-mediated reduction of NAc SCN1b had no significant effects on the acquisition or stable intake of heroin self-administration (Fig. 3*B*,*C*), operant discrimination of the drug-paired and unpaired levers (Fig. 3*D*), or lever pressing during time-out periods (Fig. 3*E*), indicating that NAc SCN1b is not required for normal active heroin self-administration behavior in male rats. Following 1 week of forced abstinence in the home cage, we observed no effects of NAc shSCN1b on context-associated heroin-seeking (i.e., the first day of responding under extinction conditions) or extinction learning behavior (Fig. 3*F*). However, after the animals reached extinction criteria, we observed that rats with AAV2-shSCN1b showed a greater number of presses of the formerly drug-paired lever following presentation of the conditioned cues (Fig. 1*G*), suggesting that NAc SCN1b functions to limit the strength of drug–cue associations and cue-reinstated heroin seeking. These results led us to hypothesize that the downregulation of NAc SCN1b by heroin self-administration (Fig. 1*D*) might be a long-lasting effect. To test this hypothesis, we allowed a new set of rats to self-administer heroin, similar to Figure 1, but instead of harvesting NAc tissue immediately after the final 3 h heroin self-administration session ended (Fig. 3*H*,*I*), we allowed the rats to go through a 7 d home-cage abstinence period and 6 d of extinction training (Fig. 3*J*). We then harvested NAc tissue the following day (i.e., when the rats would typically go into cue-reinstated seeking test; Fig. 3*A*). In contrast to Figure 1*D*, we found no changes in NAc SCN1b protein levels following 2 weeks of abstinence and extinction training (Fig. 3*K*), indicating that NAc SCN1b protein levels return to baseline levels following abstinence and extinction training. These data show that the downregulation of SCN1b is transient and suggest that reduced SCN1b during acquisition of heroin self-administration supports the formation of drug–cue associations.

## Discussion

In this study, we examined the regulation and role of the voltage-gated sodium channel auxiliary subunit, SCN1b, in NAc MSNs as it relates to heroin self-administration and cue-reinstated heroin seeking. SCN1b is expressed in NAc neurons, including D1- and D2-MSNs and multiple interneuron types (Saunders et al., 2018). We found that heroin self-administered, but not experimenter-administered, heroin injections caused a significant reduction in SCN1b protein levels in the NAc. We mimicked this effect in drug-naive rats using viral-mediated expression of shSCN1b in the NAc of adult male rats, and this produced a significant increase in intrinsic excitability in NAc MSNs. This is similar to previous findings that SCN1b null neurons are hyperexcitable (Patino et al., 2009; Lopez-Santiago et al., 2011; Brackenbury et al., 2013; Calhoun and Isom, 2014). We observed a lower depolarization threshold for MSN action potential generation, suggesting that endogenous SCN1b functions to limit MSN intrinsic excitability. However, we did note significant changes in the kinetics of the action potential, including an increase in rise-time kinetics and peak voltage change. In contrast, AAV2-shSCN1b had no apparent effects on evoked glutamatergic synaptic transmission, AMPA/NMDA ratio, AMPA/NMDA activation kinetics, or presynaptic short-term plasticity of glutamate release onto NAc MSNs. Interestingly, despite heroin self-administration causing a reduction in NAc SCN1b protein, RNAi-mediated reduction of SCN1b in NAc MSNs did not alter any assessed parameter of heroin self-administration (i.e., drug intake, operant discrimination, time-out responding) or extinction learning, but it instead significantly increased cue-reinstated heroin seeking. Taken together, our data suggest that heroin self-administration reduces SCN1b levels in NAc MSNs, which increases MSN intrinsic excitability during heroin self-administration, but this supports future opioid cue reactivity and drug seeking. In support of this idea, we recently observed that reducing NAc MSN excitability via HDAC5 overexpression was associated with decreased cue-reinstated heroin or cocaine seeking (Taniguchi et al., 2017; Anderson et al., 2023), suggesting that modulation of NAc MSN intrinsic excitability bidirectionally influences relapse-related drug seeking. As such, targeting ion channel modulators, like SCN1b, might have therapeutic potential for mitigating opioid–cue reactivity and relapse vulnerability.

While our findings reveal a role for SCN1b in NAc MSN intrinsic excitability and cued heroin seeking, there are many questions that remain for future investigation. For example, it's not clear if SCN1b's influence on cued heroin seeking is due to its modulation of intrinsic excitability or whether SCN1b might have another, different function in MSNs as well. If the effect of SCN1b on heroin seeking is directly related to its modulation of MSN excitability, which seems likely, then it will be interesting to determine whether SCN1b functions during heroin self-administration acquisition, like we detected for HDAC5 (Anderson et al., 2023), to modulate formation and/or stability of drug–cue associations or whether it might augment cued heroin seeking by increasing NAc MSN activation during the active reinstatement test. Of note, the viral-mediated knockdown of SCN1b occurs throughout all phases of heroin self-administration, extinction, and reinstatement tests, so SCN1b could modulate cued heroin seeking through a function in some or even all phases. Our findings that NAc SCN1b levels return to normal following abstinence and extinction suggest that SCN1b plays a role during the self-administration phase, but not during the reinstatement phase. Further studies examining the effects of active opioid self-administration, extinction, and reinstatement on NAc intrinsic excitability should be performed in the future to unravel these possibilities. These data suggest that opioid-induced changes that occur during the self-administration phase establish future reinstatement behavior, despite these changes returning to baseline. Since we observed no change in SCN1b protein at the time of CueRN, we would expect no changes in intrinsic excitability at this time; however, our behavioral findings suggest that these excitability changes were instrumental to future relapse-like behavior. Had this opioid-induced change in SCN1b protein remained present at the end of extinction, then this would have suggested a direct mechanism for the increased cue-induced seeking we observed. However, since SCN1b protein returned to baseline levels, our findings instead suggest that SCN1b is producing changes at the time of heroin self-administration to increase future opioid seeking. This could be tested with several future experiments. First, one could temporally limit the downregulation of SCN1b by expressing a short-lasting shRNA to SCN1b via a herpes simplex viral vector. This would reduce SCN1b for only ∼8–10 d (Hamilton et al., 2018) but allow the SCN1b levels to return to baseline during extinction and CueRN testing. We predict that this would produce an increase in CueRN. Second, one could also create a cre-dependent AAV-shRNA viral vector that can be turned off when combined with cre. By first knocking down SCN1b during the heroin self-administration phase, we could then restore normal levels of SCN1b by infusing an AAV-cre virus into the NAc to negate viral expression. We predict these rats would also still have increased CueRN behavior. Third, we could also knock down SCN1b following the completion of active heroin self-administration like in our previous publication on HDAC5 (Anderson et al., 2023). As in our previous study, we would predict that SCN1b knockdown after this self-administration period would have no effect on CueRN behavior as the temporal window would be closed. Future studies like these will be important to dissect the temporal functions of SCN1b, which could have important therapeutic implications.

Together, these data support a model where opioid-induced changes in intrinsic excitability during active opioid use lead to eventual long-lasting changes in opioid-seeking behavior, despite the return of these changes in SCN1b to baseline levels 2 weeks after the last self-administration event. For example, perhaps SCN1b downregulation is a necessary part of the process of forming new neuronal pathways that eventually lead to opioid-seeking behavior, and our manipulation of SCN1b has enhanced the formation of these maladaptive pathways. An alternative interpretation is that since these SCN1b levels return to baseline, they are inconsequential to the reinstatement behavior, perhaps due to some off-target effect of our viral manipulation strategy. In either case, these data show that reduction of NAc SCN1b is sufficient to increase cue-induced opioid seeking.

In this study, we observed that reducing SCN1b levels was sufficient to increase MSN intrinsic excitability, action potential rise-time, and amplitude. These SCN1b-specific effects mimic some opioid-induced increases in excitability previously reported. One study showed that repeated opioid administration with a 24 h withdrawal period increased NAc D2-MSN, but not D1-MSN, excitability (McDevitt et al., 2019). Another study found that repeated opioid administration reduced rheobase selectively in moderately excitable “Type II” NAc MSNs after 10–14 d of withdrawal (Wu et al., 2012; Wu et al., 2013). However, in contrast to these two studies, others have reported either no opioid-induced changes (Lefevre et al., 2023) or a decrease in excitability (i.e., increased rheobase) after opioid exposure (Heng et al., 2008). Future studies will be important to understand the apparent inconsistencies in these studies and to examine the cell-type–specific changes (e.g., D1- vs D2-MSNs) produced by volitional versus experimenter-delivered opioids at various phases of opioid use and withdrawal and the potential interaction with changes in SCN1b.

SCN1b has been linked previously to homeostatic intrinsic plasticity (Lee et al., 2015), suggesting that chronic opioid use might cause an initial change in MSN firing that drives longer-term homeostatic alterations in SCN1b levels and NAc MSN excitability that supports future drug–cue reactivity and relapse. In addition, several prior studies reported an interaction between SCN1b and the function of voltage-gated potassium channels, including kv1, kv1.3, kv4.2, and kv7 (Marionneau et al., 2012; Nguyen et al., 2012; Kubota et al., 2017). Interestingly, reduction of the voltage-gated sodium channel SCN2A (aka NaV1.2) increased intrinsic excitability in both striatal neurons (Zhang et al., 2021) and cortical neurons (Spratt et al., 2021; Zhang et al., 2021), and in both of these studies, the SCN2A-dependent changes in excitability were linked indirectly to altered potassium channel functions. Further investigation will be critical to determine if SCN1b influences NAc potassium channels similar to other brain areas.

Our results here suggest that increased NAc intrinsic excitability during active heroin self-administration augments future cue-induced drug seeking. However, other reports suggest that this may not necessarily be a common feature of all addictive substances. Unlike several reports for opioids (Wu et al., 2012; Wu et al., 2013; McDevitt et al., 2019), chronic cocaine exposure reduces NAc MSN intrinsic excitability (Kourrich and Thomas, 2009; Mu et al., 2010; Kourrich et al., 2015; Ribeiro et al., 2018). However, it should be noted that cocaine increases intrinsic excitability in NAc core MSNs but decreases it in NAc shell MSNs (Kourrich and Thomas, 2009). It will be interesting in the future to determine if cocaine self-administration regulates SCN1b protein levels. Moreover, in our study here, we targeted both the medial NAc core and shell regions with our shSCN1b manipulation, so future studies will be important to determine if SCN1b's modulation of cued heroin seeking is due to its role in the NAc core or shell or both. Based on the prior literature, we suspect that its role in cued heroin seeking involves the NAc core, which has a well-documented role in cue-reinstated heroin seeking (Fuchs et al., 2004). Since NAc MSNs have different electrophysiological properties depending on core versus shell location (Ma et al., 2012) and since addictive drugs can have opposite effects on MSN intrinsic excitability in the shell and core (Kourrich and Thomas, 2009; Kourrich et al., 2015), it will be informative in the future to repeat these studies in NAc core versus shell.

Together our findings reveal that a history of heroin self-administration reduces NAc SCN1b levels and that SCN1b limits both NAc MSN intrinsic excitability and heroin relapse-like behavior. Heroin self-administration-induced SCN1b downregulation is an opioid-induced molecular adaptation that supports opioid cue reactivity and heroin seeking, possibly by modulating NAc MSN intrinsic excitability. As mentioned, there is a myriad of interesting questions for future studies, including (1) when, and for how long, heroin self-administration downregulates Scn1b; (2) whether extinction training alters SCN1b's return to baseline following heroin self-administration; (3) in what NAc cell populations and NAc subregions does SCN1b modulate heroin seeking; (4) whether restoring or overexpressing SCN1b levels in NAc might suppress heroin-seeking behavior; (5) how SCN1b modulates MSN excitability; (6) whether the regulation and effects of SCN1b are specific to opioids versus other substances like cocaine, alcohol, or sucrose; and (7) how do these changes in NAc excitability alter signaling in other brain regions like the globus pallidus and the ventral tegmental area? However, the present findings provide a new molecular target for the potential development of OUD therapeutics and further highlight the growing appreciation that NAc MSN intrinsic excitability plays an important role in drug–cue associations and the addiction-related neuroadaptations underlying relapse vulnerability.

## Synthesis

Reviewing Editor: Sam Golden, The University of Washington Decisions are customarily a result of the Reviewing Editor and the peer reviewers coming together and discussing their recommendations until a consensus is reached. When revisions are invited, a fact-based synthesis statement explaining their decision and outlining what is needed to prepare a revision will be listed below. The following reviewer(s) agreed to reveal their identity: NONE. Note: If this manuscript was transferred from JNeurosci and a decision was made to accept the manuscript without peer review, a brief statement to this effect will instead be what is listed below.

Dear Drs. Anderson and Cowan,

After reading through the two prior rounds of reviews at JN, and the rebuttal provided with the transfer to eNeuro, I believe the manuscript is appropriate for publication without new reviews.

I do have one minor comment that may be addressed during proofing. The orientation of the histological images in Figure 1H may be confusing to readers; please consider changing their orientation so that dorsal is up.
